# Resilience and the Gut Microbiome: Insights from Chronically Socially Stressed Wild-Type Mice

**DOI:** 10.3390/microorganisms10061077

**Published:** 2022-05-24

**Authors:** Malena dos Santos Guilherme, Francesco Valeri, Jennifer Winter, Marianne B. Müller, Andreas Schwiertz, Kristina Endres

**Affiliations:** 1 Department of Psychiatry and Psychotherapy, University Medical Center, Johannes Gutenberg University Mainz, 55131 Mainz, Germany; malena-guilherme@gmx.de (M.d.S.G.); francescovaleri47@gmail.com (F.V.); marianne.mueller@lir-mainz.de (M.B.M.); 2 Institute for Human Genetics, University Medical Center, Johannes Gutenberg University Mainz, 55131 Mainz, Germany; jewinter@uni-mainz.de; 3 Leibniz Institute for Resilience Research (LIR), 55122 Mainz, Germany; 4 MVZ Institute fuer Mikrooekologie GmbH, 35745 Herborn, Germany; andreas.schwiertz@mikrooek.de

**Keywords:** chronic social stress, resilience, susceptibility, mouse model, microbiome, antibiotics, probiotics

## Abstract

The microbiome is an important player within physiological homeostasis of the body but also in pathophysiological derailments. Chronic social stress is a challenge to the organism, which results in psychological illnesses such as depression in some individuals and can be counterbalanced by others, namely resilient individuals. In this study, we wanted to elucidate the potential contribution of the microbiome to promote resilience. Male mice were subjected to the classical chronic social defeat paradigm. Defeated or undefeated mice were either controls (receiving normal drinking water) or pre-treated with antibiotics or probiotics. Following social defeat, resilient behavior was assessed by means of the social interaction test. Neither depletion nor probiotic-shifted alteration of the microbiome influenced stress-associated behavioral outcomes. Nevertheless, clear changes in microbiota composition due to the defeat stress were observed such as elevated *Bacteroides* spp. This stress-induced increase in *Bacteroides* in male mice could be confirmed in a related social stress paradigm (instable social hierarchy) in females. This indicates that while manipulation of the microbiome via the antibiotics- and probiotics-treatment regime used here has no direct impact on modulating individual stress susceptibility in rodents, it clearly affects the microbiome in the second line and in a sex-independent manner regarding *Bacteroides*.

## 1. Introduction

The emotional behavior and well-being of an organism seem not solely to rely on the psychological traits of the host itself. While the impact of environmental factors has been overly accepted, the impact of the microbial commensals or pathological microbes has long been neglected. Within the last decade, the effects of the microbiome on the host’s metabolism, and thereby on their behavior, have been put into focus. Antibiotics-derived depletion of the gut microbiome has been found to act, for example, on neurological disorders modelled in rodent animals such as Alzheimer’s disease (AD; [1,2]). Moreover, for human patients as well, correlations with certain taxa in relation to Parkinson’s disease (PD), AD or autistic phenotypes and many more disorders have been described (e.g., meta-analysis regarding PD by [3] and regarding AD by [4]; data on autism reviewed in [5]). Therefore, it seems plausible that the microbiome in general might affect the cognitive and psychological health of its host—not only in the diseased stage but also in the maintenance and stability of the psyche.

The outcome as to whether external threats will lead to disease development or whether an individual stays healthy despite all adversities depends on the resilience of the individual [6]. Many host-allocated factors that regulate or were attributed to resilience such as the inflammasome [7], neurotrophic hippocampal factors [8] or physical activity [9] have already been identified. However, it seems highly plausible that the microbial community of the gastrointestinal system might be one of the factors to be considered. As the stress response tremendously affects various physiological properties of the host such as the hypothalamus-pituitary-adrenal (HPA) system, it in turn influences the microbiome. Already in 2016, the chronic social defeat paradigm has been used in male C57BL6/J mice to investigate changes in the fecal microbiome [10]: reduced richness and diversity was found in male mice subjected to 10 days of defeat by an aggressor mouse from the CD-1 strain.

Increased corticosterone levels/adrenal hypertrophy [11] altered α-defensin secretion by Paneth cells [12], or changes within the mucosa such as lectin presentation [13] might elicit effects on the microbiome. The host, however, might be influenced by its microbiome via production of pro-inflammatory cytokines (IL-1, IL-6) produced in response to certain strains of gut bacteria, which then can activate the HPA axis [14]. Moreover, gene expression of the pituitary gland and the adrenal gland has been shown to be reactive to microbial commensals when comparing SPF and germ-free BALB/c mice [15]: expression of Fkbp5 (a gene involved in glucocorticoid-sensitivity) was, for example, found to be higher in the pituitary gland of SPF male mice than in that of germ-free mice. Similarly, a deprivation of the microbial community by 14 days of antibiotics treatment prevented occurrence of an anhedonia-like behavior by chronic social defeat stress (CSDS) in mice [16]. Besides these depletion approaches, substitution with single alive microorganisms, heat-killed supplements or prebiotics have been conducted. For example, oral administration of a mixture of short chain fatty acids, important metabolites of gut commensals, failed to ameliorate the CSDS-evoked changes in the social interaction score in male C57BL6/J mice [17]. In contrast, Bifidobacteria were found to be increased in resilient male mice upon CSDS, and oral intake of Bifidobacteria increased the amount of resilient individuals [18]. This gives an example of how controversial data regarding the effect of microbes on stress resistance are. This inconsistency might often be deduced to factors not directly associated with the experiment but related to the environment such as the attendant experimenter or other ongoing experiments.

Antibiotics lead to a disruption of the healthy microbial community, while probiotics are assumed to have a beneficial effect on both the host and the microorganisms within the gut [19,20]. To directly compare the influence of either a microbial depletion or a substitution with probiotic organisms and to eliminate potential confounding external factors, we herein parallel treated C57BL6/J OlaHsd male mice with an antibiotics cocktail or supplemented their drinking water with a mixture of *Lactobacillus acidophilus* and *L. rhamnosus*. Subgroups of mice underwent CSDS and living representatives of the microbial community (*Lactobacillaceae* and *Enterobacteriaceae*) were monitored, demonstrating the efficacy of the treatment. However, both manipulations did not result in a change in the social interaction, which was measured as an outcome of CSDS. Nevertheless, an increase in the *Bacteroides* spp. could be assessed when comparing defeated and non-defeated animals. For a long time, chronic social stress has been only investigated in male rodents due to the underlying behavioral task. Within recent years, models for females have also been established, allowing for investigations into their reaction to chronic social stress. The social instability paradigm that we recently published for C57BL6/J mice [8] was used here to compare the effect of CSDS in males with outcome in females: interestingly, an increase in *Bacteroides* spp. was also noted in stressed female animals, suggesting a potential common mechanism of sex in reaction to chronic social stress.

## 2. Materials and Methods

### 2.1. Animals

C57BL6/J OlaHsd mice were obtained from Envigo (Horst, The Netherlands) at the age of 3 weeks and subsequently maintained in a 12 h light/dark cycle with food (Ssniff Spezialdiäten GmbH, Soest, Germany) and water available *ad libitum* in the local facility. ARC-creERT2/+.R26CAG-LSL-Sun1-sfGFP-Myc/+ (hereby termed as Arc-sfGFP, [8]) mice were obtained via in-house breeding from the Johannes Gutenberg University (TARC). This mouse model allows specific Tamoxifen-dependent CRE-recombination under the promoter of the immediate early gene Arc in nuclei activated by stressful events, for example. The nuclei subsequently show sfGFP labelling on their membrane [21] and thus are to be identified by fluorescence microscopy. All procedures were performed in accordance with the European Communities Council Directive regarding the care and use of animals for experimental procedures and were approved by local authorities (Landesuntersuchungsamt Rheinland-Pfalz; approval number G 17-1-035 or G 17-1-021).

### 2.2. Chronic Social Stress

**Figure 1:**
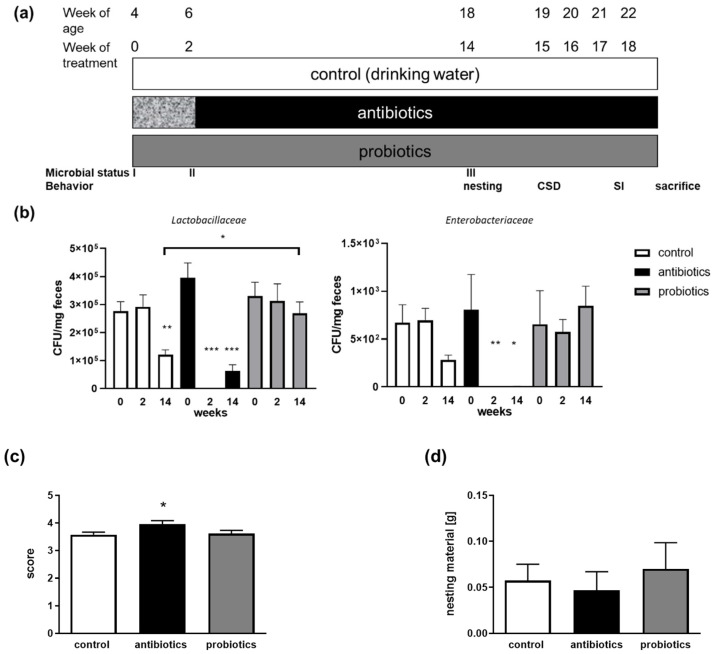
(**a**) Time schedule of treatment of the mice and testing. Three groups of mice were used, all male C57BL6/J. Control mice received normal drinking water throughout. Mice belonging to the antibiotics group were set on drinking water supplemented with an antibiotics cocktail at high (first two weeks, patterned box) or low maintenance dosage (from six weeks of age up to euthanization). Probiotics-treated mice received drinking water supplemented with probiotics. All bottles were changed twice a week. All mice were trained in the week after weaning up to the start of the experiment to use the special small-sized drinking bottles to assure sufficient consumption of water. Mice were randomly assigned to the subgroups “defeated” and “undefeated” for all treatments. (**b**) Effect of microbiome manipulation on *Lactobacillaceae* and *Enterobacteriaceae*. Fecal samples for plating on selective agar plates were taken at three different time points: before the start of treatment (0 = with the age of 4 weeks, I; 2 = with the age of 6 weeks, after two weeks of high-dosage treatment, II; 14 = with the age of 18 weeks, after 14 weeks of treatment, III). Colony forming units (CFUs) were normalized to the fecal material weight. Randomly selected mice were tested at all three time points (n = 7–21 per group, data are shown as mean + SEM). Statistics were performed using one-way ANOVA with Fisher’s LSD test (* *p* < 0.05; **, *p* < 0.01; ***, *p* < 0.001). (**c**) Well-being and hippocampal general function were assessed by scoring nest-building ability within the 14th week of treatment (mice were aged 18 weeks). (**d**) Besides the score, the unused material remaining outside of the nest was also weighed. n = 40 for controls and 30 for each treatment (to be defeated and to be undefeated mice are still integrated in one group here, as defeat was administered after the nesting test). Data are shown as mean + SEM (one eay ANOVA with Tukey’s post-test (* *p* < 0.05)).

For males, the CSDS paradigm was used as described in [22]. In brief, C57BL6/J OlaHsd intruder mice were placed in a home cage of a resident CD-1 mouse previously screened for aggressive behavior. After 2 min of social defeat, the mice were separated by a mesh. Mice were kept separated in the same cage for 24 h. The procedure was repeated for 10 consecutive days, with a new resident CD-1 mouse used daily to prevent habituation. Undefeated mice were placed in pairs in a cage separated by a grid, as were stressed mice, and were transferred into a clean cage daily. On the eleventh day, all mice were placed individually in new cages. Mice were randomized to either a control group (drinking water only), antibiotics group or probiotics group (see Figure 1a). Mice were allowed *ad libitum* access to the water bottle containing high doses of antibiotics for two weeks. Each antibiotic was dissolved in sterile water in stock concentrations, and aliquots of stocks were frozen at −20 °C. Antibiotics were mixed together in tap water at the following final concentrations (for the high dosage): Gentamicin (0.083 mg/mL), Vancomycin (0.042 mg/mL), Metronidazole (0.167 mg/mL), Neomycin (0.042 mg/mL), Ampicillin (0.083 mg/mL), Colistin (500 U/mL), Cefoperazone (0.083 mg/mL) and Kanamycin (0.25 mg/mL) (all Sigma Aldrich, Steinheim, Germany or Cayman Europe, Tallinn, Estonia). After the second week of treatment, mice were kept at a low dosage regime (1/50 of initial concentration) throughout the rest of the experiment (until the eighteenth week). Mice assigned to the vehicle control group were subjected to the same treatment protocol with tap water only (until the eighteenth week). This treatment was based on [1]. In our previous experiments conducted in Alzheimer’s disease model mice [23], we observed an effective reduction in the gut microbiome by a similar antibiotics cocktail. Probiotics-treated animals received 10^9^ CFU/mL (OptiBac for those on antibiotics, OptiBac Probiotics HQ Wren Laboratories Ltd., Hampshire, UK, contains *L. acidophilus* and *L. rhamnosus*), which resulted in elevated amounts of *Lactobacillaceae* in another mouse strain [23] and is in accordance with other published treatment approaches in rodents (for example, [24,25]). The water for all groups was exchanged two times a week. Food and water consumption was continuously monitored before the mice entered the defeat, and drinking volume per day was assessed as about 4.5 mL (4.6 mL ± 0.5 mL in the first batch of mice, for example). 

Female C57BL6/J OlaHsd and Arc-sfGFP mice were subjected to a social instability paradigm, eliciting stress as measurable by corticosterone elevation or low-frequency ultrasonic calls [8]. Briefly, mice were kept in groups of four from four weeks on, and groups were shuffled two times a week following an algorithm that prevented re-introducing known animals up to week five of the shuffling procedure. Overall, mice were stressed for seven weeks (including 14 to 15 shuffling events). Animals were analyzed either directly after the last stress application or one year after discontinuation of the stress procedures. For the latter, mice were kept in the last-achieved group constellation. Control groups were kept in stable group compositions throughout. 

Tamoxifen injection in female Arc-sfGFP mice (Supplementary Figure S1) was conducted as described in [8]. The procedure for male mice is described in [26].

### 2.3. Quantitation of Bacterial CFUs

Viable *Lactobacillaceae* and *Enterobacteriaceae*, as representative families of gut commensals, were assessed by counting colony forming units (CFUs) as described previously [27]. Voluntarily provided feces collected after 3 pm was homogenized with a hand-held device (Xenox, Fähren, Germany) in 0.9% sodium chloride and diluted appropriately. Then, 1 mL of bacterial suspension was spread on selective plates (3M Deutschland GmbH, Heidelberg, Germany) and incubated at 37 °C overnight. CFUs were counted from whole plates (*Enterobacteriaceae*) or representative sectors of plates (*Lactobacillaceae*) and normalized to fecal material weight. Feces was collected before the start of the treatment with anti- or probiotics (week 0), after two weeks and before the start of the CSDS paradigm (14 weeks) in males.

### 2.4. qPCR for Quantitation of Selected Bacteria

For females, collection of feces took place after 7 weeks of stress administration with euthanization (age of 13 weeks) or one year after this. For males, collection of feces was conducted with euthanization (22 weeks of age). Fecal samples were analyzed at the MVZ Institut fuer Mikrooekologie GmbH (Herborn, Germany) as described before [23]. Standard curves were produced using the appropriate reference organism to quantify qPCR values into the number of bacteria per gram (wet weight). Values obtained were normalized to the mean of the total numbers of sequences obtained.

### 2.5. Behavioral Tests

The nesting test has been described previously [28]. In brief, mice were habituated to a specific nesting material from paper stripes, and the nest built after one night of deprivation to nest material was scored. Mice were single-caged three to five days before the nesting test to allow single-animal read-outs.

Social interaction was assessed in male mice as described before [29]. Briefly, the time spent within a defined region around an empty cylinder and the time spent with a male conspecific from the aggressor strain (CD-1) were measured. SI score was calculated from the ratio of time in the zone with the unknown mouse to the time in the zone without the mouse and is given as percentage.

### 2.6. Sacrifice and Tissue Preparation

Animals were anesthetized by isoflurane inhalation and euthanized by decapitation. Tissue specimens were collected in pre-weighed tubes and the weight per body weight was calculated. Serum was derived from truncal blood after a minimal clotting time of 45 min by double centrifugation (1680× *g*, 10 min, 10 °C and 15,680× *g*, 10 min, 10 °C). Serum corticosterone levels were assessed as described previously [8].To assess the activated nuclei of enteric neurons within the myenteric plexus, gut specimen from Arc-sfGFP mice were collected upon euthanization, and the myenteric plexus attached to the longitudinal muscle was separated from the circular muscle (designated LMMP). LMMPs were spread on cover slips in a physiological salt solution and directly investigated using a Zoe Fluorescent Cell Imager (Bio-Rad, Feldkirchen, Germany) microscope. Four LMMPs from ileum sections were obtained from each mouse, and a total of four images were acquired from each section. Unspecific background staining (rolling ball radius: 50 pixel) was subtracted from every image using ImageJ software (https://imagej.nih.gov/ij/, Rasband, WS, USA). sfGFP-positive neurons were counted in three defined and randomized squares areas (888 pixels for width and height) for every picture (Supplementary Figure S1a). The number of sfGFP-positive neurons contained in each area was analyzed by ImageJ software.

### 2.7. Statistical Analyses

Statistical significance was determined by Student’s *t*-test with additional Welsh correction if needed or by one-way ANOVA with Fisher’s LSD post-hoc test (Graph Pad Prism 6 and 8). A *p*-value of <0.05 was assigned as statistically significant. Data are presented as means + SEM or means with minimum and maximum values as indicated. Outliers were removed if identified by the ROUT method with a Q of 1% (GraphPad Prism, GraphPad Software, San Diego, CA, USA).

## 3. Results

We aimed at investigating the potential mutual influence of the microbiome and perception of chronic social stress in C57BL6/J mice. For this purpose, on the one hand, we analyzed the microbiome within non-manipulated mice with regard to the effect of stress (designated “control”, received normal drinking water, Figure 1). On the other hand, we manipulated the microbiome by administering antibiotics and probiotics via the drinking water before and along the social defeat and read-out.

### 3.1. Treatment to Modify the Gut Microbiome

Mice were either treated with a mixture of two probiotics (*L. acidophilus* and *L. rhamnosus*) or with two weeks of high-dosage antibiotics, with subsequent reduced maintenance dosages (Figure 1a). The efficacy of the microbiome manipulation was analyzed by plating exemplary bacterial families on selective agar. *Lactobacillaceae* and *Enterobacteriaceae* were chosen for their known role as putative positively and negatively annotated commensals of the gut [30,31,32]. The start amounts of the respective viable bacteria were not different in all three groups of mice (Figure 1b). However, the antibiotics treatment severely reduced the amount of Lactobacillaceae after 2 weeks of high-dosage treatment and even 14 weeks later. In the reduced maintenance dosage, there remained a significantly decreased number of viable counts. For Enterobacteriaceae, the treatment was comparably efficiant with nearly no CFU detectable in feces from treated mice. For the probiotics-treated animals, only a difference in Lactobacillaceae was obtained at the third test point (14 weeks of age): here, higher numbers of bacteria occurred as compared to the control mice.

To ascertain that the well-being of the animals and behavior per se was not affected, we also assessed their nest-building capability (Figure 1c,d). Unused nesting material was comparable between all three groups. Antibiotics-treated mice showed a slightly increased nesting score as compared to both other groups.

### 3.2. Investigation of Social Interaction

Avoidance behavior towards a conspecific of the aggressor strain is a typical outcome following chronic social defeat stress in male mice. Specifically, the defeated C57BL/6J mice are exposed to a novel CD1 male mouse that is placed below a mesh, thus mimicking confrontation with a conspecific of the aggressor strain that used to trigger social defeat sessions before (retired breeders). Outcomes in this social interaction test typically display considerable heterogeneity, with some of the defeated mice still showing a high degree of exploration towards the CD1, and thus will approach the CD1 mouse while other defeated mice will avoid contact. This behavior has been designated as being resilient or susceptible [29]. The resilience of mice is defined by a percentage SI score (time spent in the interaction zone with a social object/time spent in the interaction zone without a social object) above 100%. Animals with a score below 100% are defined as being susceptible.

**Figure 2:**
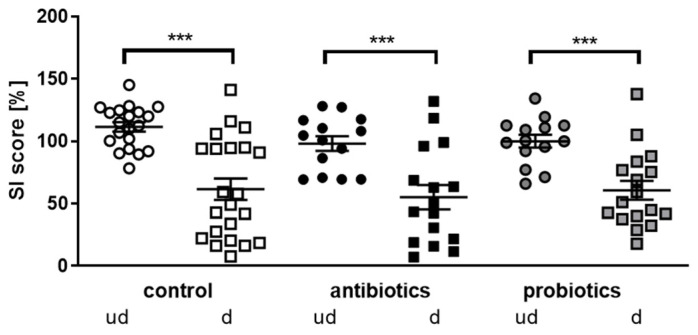
Social interaction of microbiome-manipulated male mice. Mice that underwent antibiotics or probiotics treatment or served as controls were either let undefeated (ud) or received defeat (d) during 10 consecutive days. One week after the CSDS, social interaction was assessed by presenting an unknown male CSD1 mouse. The SI score is calculated as the percentage time spent in the interaction zone around the Plexiglas cylinder containing the CD1 mouse divided by the time spent in the same zone during the habituation (plexiglas cylinder containing no other mouse). Statistics were performed using one-way ANOVA with Fisher’s LSD post-test (*** *p* < 0.001; n = 20, 22, 14, 16, 14, 17). Data are shown as mean + SEM.

When measuring movement during the habituation phase or within the social interaction phase, animals of all three groups were indistinguishable and also not to be distinguished from undefeated mice (e.g., distance moved during the habituation phase for the batch of undefeated controls: 1019 cm; defeated controls: 1122 cm; antibiotics-treated mice: 1067 cm; probiotics-treated mice: 976 cm; *p* > 0.89 for all comparisons). Thus, locomotive properties were not affected due to the applied CSDS. However, a clear difference between defeated and undefeated mice was observed in all three treatment groups concerning social interaction (Figure 2). Defeated mice revealed a significantly decreased social interaction score, proving the efficacy of the respective paradigm. However, no effect of the microbial manipulation could be observed.

### 3.3. Physiological Parameters of Mice after Microbial Manipulation and Social Defeat

**Figure 3:**
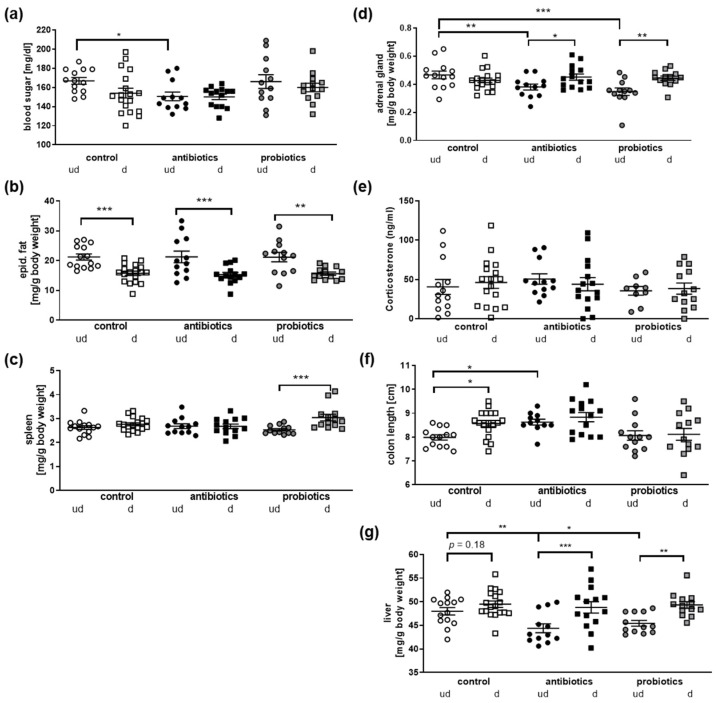
Physiological parameters in mice after CSDS. Mice that underwent antibiotics or probiotics treatment or served as controls derived from defeated (d) or undefeated (ud) groups were euthanized after assessing social interaction. (**a**) Blood sugar levels were measured using truncal blood directly upon euthanization. (**b**) Epididymal fat deposits were extracted and weighed. Data are shown after normalization to body weight. (**c**) Spleen was dissected and relative weight calculated. (**d**) Adrenal glands were extracted and weight assessed. Shown are values normalized to body weight. (**e**) Corticosterone serum levels were measured by HPLC method. (**f**) Colon length was measured directly during dissection, and liver weight assessed and normalized to body weight (**g**). All values present mean with SEM. All values were included if not identified as outliers (Q of 1%). Statistical analysis was performed by one-way ANOVA with Fisher’s LSD test (* *p* < 0.05, ** *p* < 0.01, *** *p* < 0.001, n = 10–18 for all groups).

As the anti- and probiotics treatment did not affect responses to chronic social defeat, we investigated the systemic impact of each condition at or after euthanization (Figure 3). Blood sugar was decreased in antibiotics-treated mice, as already observed before (Figure 3a; [2]). The amount of epididymal fat was decreased in all defeated animals in comparison to their respective controls (Figure 3b). This might be due to the increased energy expenditure during the encounters. Spleen weight was mainly unaffected except in the probiotics defeated mice, where a slight weight increase occurred (Figure 3c). As social stress severely impacts the HPA axis (e.g., [33]), adrenal gland weight was assessed (Figure 3d): here, the weights were indistinguishable for the control and treated mouse groups (defeated, undefeated). However, both, the antibiotics as well as the probiotics groups revealed statistically significant decreases in adrenal weight due to the social defeat. This might suggest that reaction to stress should be different in the three treatment groups. Nevertheless, adrenal weights in all three groups after defeat were comparable. This also is conclusive with the observation that corticosterone serum levels were also not to be distinguished between all conditions (Figure 3e). Here, however, it has to be acknowledged that euthanization took place nearly one week after the last defeat. Thus, corticosterone levels might have already returned back to normal. The slight increase in the defeated control group, for example, did not reach statistical significance (Figure 3e; *p* = 0.59). However, resilient control mice displayed a significantly lower level as compared to susceptible control mice (37.16 ng/mL versus 72.85 ng/mL, n = 6 per group; *p* = 0.04).

**Figure 4:**
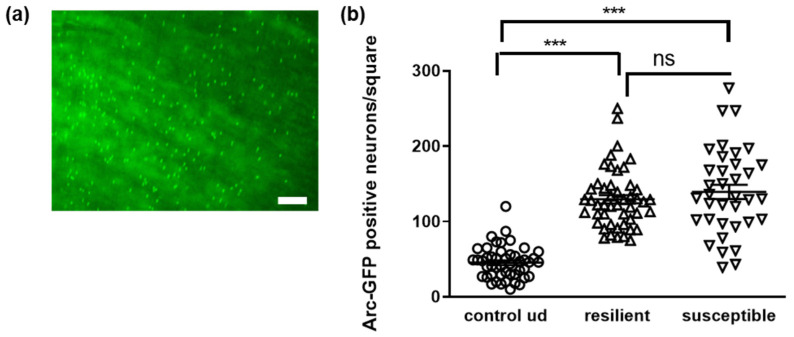
Activation of enteric neurons during social interaction testing after chronic social defeat treatment. Male Arc-sfGFP mice were treated as described for C57BL6/J mice (see Figure 1a). Tamoxifen was injected 5 h before social interaction to label activated neurons [26]. (**a**) Arc-expressing, activated neurons were visualized by their GFP signal under the fluorescent microscope in LMMPs from the ileum of defeated and undefeated control mice. Scale bar: 100 µm. (**b**) Stained cells were counted from a defined area for each animal (see also Supplementary Figure S1). Statistical analysis was conducted by one-way ANOVA (Tukey’s multiple comparison test; *** *p* < 0.001, n = 48 for undefeated and 48/36 for defeated animals, derived from four (control/resilient) and three (susceptible) mice per group).

Finally, we measured the colon length and observed that antibiotics treatment elongated the organ, which coincides with previous reports (Figure 3f, [34]). More interestingly, a prolonged colon length was found when comparing undefeated and defeated control mice, suggesting a direct influence of the social stress on the intestinal system. This is also in line with the effect observed on the liver (Figure 3g): in all three treatment groups, liver weight increased due to the defeat in comparison to undefeated animals, which reached significance in the pro- and antibiotics group. Interestingly, the liver weight of undefeated, antibiotics- and probiotics-treated animals was lower than the weight observed from controls. Such hepatic weight reduction has been especially reported in the context of induced fatty livers [35,36,37]. In sum, this shows that the gastrointestinal system is tremendously affected by the chronic social defeat paradigm, probably by the gut–liver–brain axis (for a recent review on the role of this organ axis in diseases, see [38]). Thus, it might be assumed that the perception and reaction to the stressful events within the CNS are transferred via the vagus nerve or soluble mediators towards the intestines and associated organs such as the liver. Nevertheless, the gut inherits an extended neuronal network itself that might directly respond to the aversive situation. To prove if the stress experience might also affect the enteric nervous system, we made use of a transgenic mouse model with which we previously confirmed a similar reaction to social stress, at least in female individuals (see [8]). These mice allow for the trapping of activated neurons by means of Arc-promoter-driven GFP expression subsequent to Tamoxifen administration [21]. Mice were euthanized after the respective stress treatment paradigm, and myenteric plexus layers with longitudinal muscle (LMMP) were prepared. The amount of activated, GFP-positive cell counts was assessed by fluorescence microscopy (Figure 4). 

**Figure 5:**
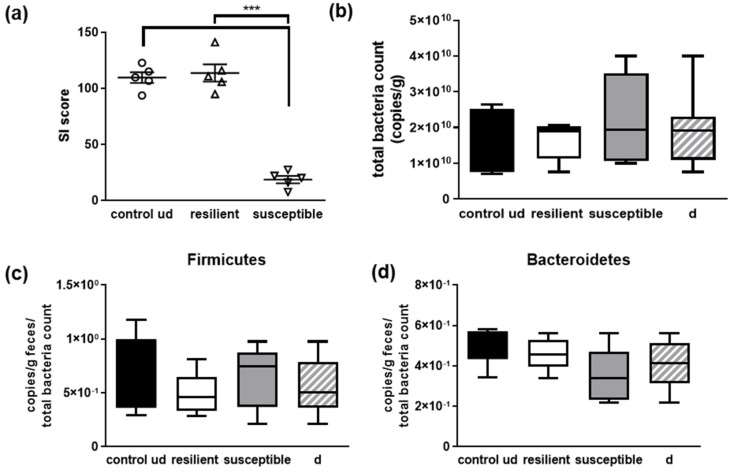
General parameters of the microbiome of defeated and undefeated mice. (**a**) Mice were selected from the group of control mice (Figure 2) due to their achieved SI score, with resilient mice having a comparably high score and susceptible mice characterized by a low score. (**b**) The bacterial count per g of feces was measured as well as the relative amount of Firmicutes (**c**) and Bacteroidetes (**d**) via qPCR. Data are shown as means and min to max. Statistics were performed using one-way ANOVA with Fisher’s LSD test (*** *p* < 0.001). None of the comparisons was statistically significant (n = 5 per group; undefeated controls = ud; defeated controls = d (mean of resilient and susceptible, n = 10)).

The immediate early gene Arc is also expressed in enteric neurons, and the activity of its promoter led to a strong and clearly visible GFP signal in LMMPs derived from ileum (Figure 4a). Mice that underwent chronic social stress before the assessment of social interaction indeed indicated a higher number of activated neurons (Figure 4b) in the gut, with no differences between resilient and susceptible animals. Thus, chronic social stress affects the intestinal system not only via CNS responses to the paradigm, but might also directly involve the neuronal system of the gut. All together, these findings strongly support that changes in the microbial community of the gut, the microbiome, might also be expected. If this might lead to a differentiated signature between resilient and susceptible individuals, we next investigated this by using respective subgroups of the control groups (see Figure 5a).

### 3.4. Microbial Signature of Defeated Mice, Depending on Being Resilient or Susceptible

From the control group of C57BL6/J mice (Figure 2), five mice each were chosen for further downstream analysis: undefeated controls and resilient as well as susceptible individuals from the defeated group. All of them were selected based on their performance in the social interaction test. Undefeated controls were selected by providing scores most closely to the mean of their group, resilient animals were selected by the highest SI score within the group and susceptible animals by the lowest values (Figure 5a). First, we measured total bacterial counts and could not observe differences between the undefeated controls and defeated individuals from the extreme groups as well as between resilient and susceptible animals (Figure 5b). Similar results were obtained for the two phyla that constitute the predominant groups of murine gut bacteria—Firmicutes and Bacteroidetes ([39]; Figure 5c,d). Slight increases in Firmicutes in susceptible animals occurred as well as decreases in Bacteroidetes in comparison to the resilient mice; however, this did not reach statistical significance (*p* = 0.37 and 0.13).

**Figure 6:**
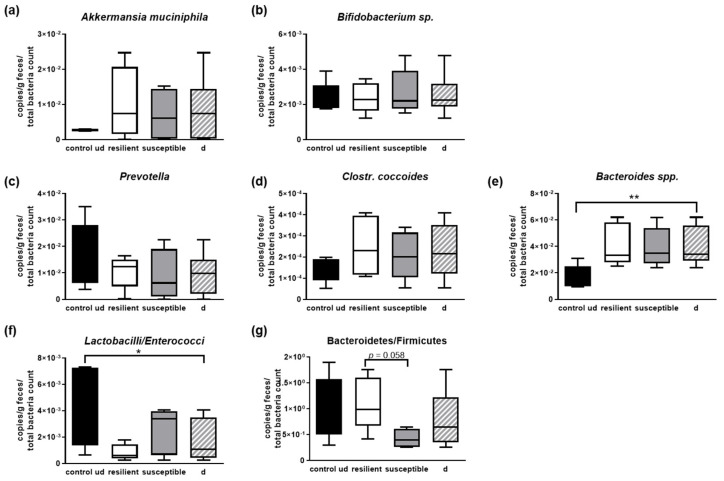
Analysis of microbial composition of feces derived from defeated and undefeated male mice. (**a**) The feces of the five selected animals per group (see Figure 5a) was analyzed in more detail via qPCR regarding representative microbial commensals: (**a**) *Akkermansia muciniphila*, (**b**) *Bifidobacterium* sp., (**c**) *Bacteroides* group, (**d**) *Prevotella*, (**e**) *Clostridium coccoides*, (**f**) ratio of *Lactobacilli* to *Enterococci* and (**g**) Bacteroidetes–Firmicutes ratio. Data are shown with means and min to max. Statistics were performed using one-way Anova with Fisher’s LSD test (* *p* < 0.05, ** *p* < 0.01). None of the comparisons were statistically significant (n = 5 per group; undefeated controls = ud; defeated controls = d (mean of resilient and susceptible, n = 10)).

Subsequently, we performed a more detailed analysis regarding different single species or bacterial groups (Figure 6). The amount of *Akkermansia muciniphila* and undefined Bifidobacteria were not to be distinguished between the different groups even if *A. muciniphila* displayed a trend towards elevation in stressed individuals (Figure 6a,b), as well as *Prevotella* and *Clostridium coccoides* (Figure 6c,d). On the contrary, Bacteroides were increased in all groups as compared to the undefeated controls (Figure 6e). To analyze important opponents in the microbial community, we also measured *Lactobacilli–Enterococci* and Bacteroidetes–Firmicutes ratios. The latter one especially has been investigated in various human diseases and in rodent models for those diseases or pathological conditions (critically reviewed in [40] for obesity). The ratio of *Lactobacilli*–*Enterococci* decreased due to the defeat stress (Figure 6f). Finally, while the groups of defeated and undefeated mice were indistinguishable regarding the Bacteroidetes–Firmicutes ratio, an increase between individuals characterized as resilient in comparison to those designated susceptible occurred (Figure 6g).

### 3.5. Changes in Microbiota in Females Subjected to a Chronic Social Stress Paradigm

The paradigm used to subject males to CSDS contains a certain amount of physical attacks with subsequent wounding. This might influence the immune system and thus the microbial commensals [41,42]—an aspect that might be lacking when applying certain paradigms of chronic social stress to females that do not contain physical components. Moreover, the microbiome has been shown to exert a strong sex-dependency per se in humans but also rodents [43]. Therefore, we next wanted to investigate if the observed influence of chronic social stress also elicits similar changes in female wild-type mice. For female mice, we chose the social instability paradigm that successfully leads to physiological signs of stress in female mice [8]. Fluorescence microscopy of the myenteric plexus from Arc-sfGFP mice confirmed that the enteric nervous system was similarly activated in females as in males due to social stress (see Supplementary Figure S1). However, the number of activated neurons was already higher in control females as compared to undefeated males (females: 78 ± 9 versus male: 45 ± 3; *p* = 0.017), pointing at a certain sex dimorphism in the enteric nervous system.

**Figure 7:**
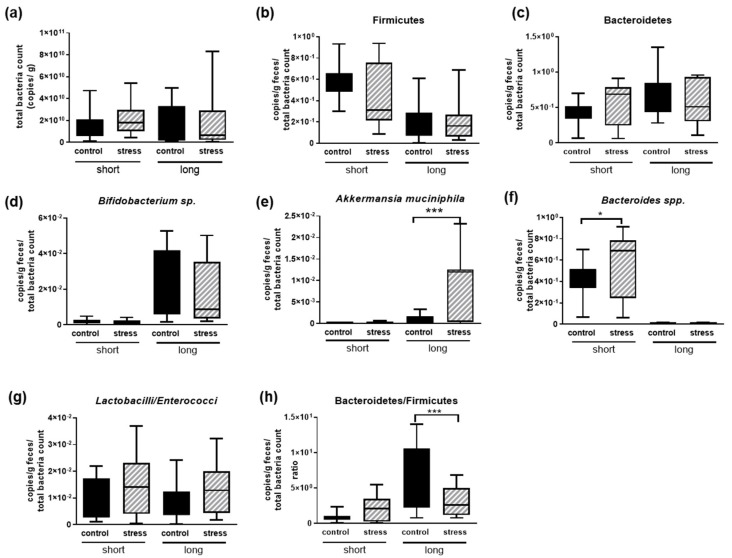
Analysis of microbial composition of feces derived from C57BL6/J OlaHsd female mice after chronic social stress. Feces of animals were collected (n = 12 per group) and analyzed for the following bacterial species and groups: (**a**) total bacteria, (**b**) Firmicutes, (**c**) Bacteroidetes, (**d**) *Bifidobacterium* sp., (**e**) *A. muciniphila*, (**f**) *Bacteroidetes* spp. (**g**) ratio of *Lactobacilli* to *Enterococci* and (**h**) Bacteroidetes–Firmicutes ratio. Data are shown with means and min to max. Statistics were performed using one-way ANOVA with Fisher’s LSD test (* *p* < 0.05; *** *p* < 0.001). “Short” indicates animal groups that were directly analyzed after having experienced the stress, and “long” indicates groups of animals that were maintained for one year in stable group compositions after the last stress-inducing shuffling of groups.

Total counts of bacteria as well as the Firmicutes, Bacteroidetes and *Bifidobacterium* sp. amounts were indistinguishable between stressed and control female mice (Figure 7a–d), as seen before for males. For the females, we also conducted a long-term follow-up for 1 year after the last stressful event and could demonstrate that some indicators of stress still remained while others vanished (“long” in Figure 7; [8]). However, no long-term effects regarding these first three microbiota parameters occurred. For *A. muciniphila*, directly after the stress, a slight increase was obtained that did not reach statistical significance; however, one year after the stress, females that were derived from stressed groups showed significantly elevated amounts when compared to animals that did not experience social stress (Figure 7e). *Bacteroides* spp. that were increased in males after social stress (Figure 6e) also revealed increased amounts in stressed females (Figure 7f). This effect was completely abolished one year after the stressful experience.

While male mice displayed a decreased *Lactobacilli*–*Enterococci* ratio due to being exposed to the defeat paradigm (Figure 6f), females showed no differences—even directly or one year after being stressed (Figure 7g). Comparing in general the Bacteroidets–Firmicutes ratio between the control and stressed animals, females showed no effect, similar to males (Figure 6g and Figure 7h). However, within the females, a delayed effect was observed one year after the stress: while the ratio increased in control animals along the age-gaining process, it remained rather small in animals that had the stress experience in their adolescence/early adulthood (Figure 7h, “long”).

## 4. Discussion

In our study, the directionality of gut microbiome manipulation had no significant impact on modulating chronic-social-stress-related behavioral outcomes in a social interaction test on male mice: whether the animals received antibiotics or probiotics did not affect their SI score, even if the efficacy of the microbiome manipulation was evident as measured by the cultivatable exemplary families *Lactobacillaceae* and *Enterobacteriaceae*. The number of resilient males in the control group was comparably low with 18%, as mostly 20% or more animals are identified [8,29]. This might be due to the comparably limited number of investigated animals in total, as mostly higher numbers are needed to precisely determine the ratio of resilient versus susceptible individuals. Nevertheless, a beneficial effect, i.e., elevated numbers of resilient mice, was not observed. A previous report on antibiotics treatment described such positive effects on CSDS in male mice; however anhedonia was the only behavioral outcome investigated, failing to include social interaction subsequent to the chronic social stress exposure [16]. Other investigations involved bacterial strains that might be seen as close relatives to *L. rhamnosus* and *L. acidophilus*, the two probiotic strains we used in our study. Four weeks of oral treatment with *L. rhamnosus* (JB-1), for example, decreased anxiety in C57BL6/J male mice (Light–Dark Test; [44]) and restored sociability towards a conspecific. However, interestingly, this treatment was not able to ameliorate avoidance behavior towards CD-1 used as the aggressors, and thus coincides with our findings. Treatment for 28 days with *L. reuteri* could restore some changesinf the microbiome as a result of CSDS and also influence the serotonine metabolism [45]. Contra intuition, it reduced acetate and total SCFAs in the stressed mice while treatment with butyrate, for example, has been shown to have rather anti-depressant effect in rodents before [46,47]. Such discrepancies might also rely on the dosage of the administered probiotics. A combined treatment with *L. helveticus* R0052 and *Bifidobacterium longum* R0175 (Cerebiome, Lallemand) revealed similar outcome for high-dose and controls while low-dose-treated animals showed increased susceptibility to social stress in Syrian hamsters [48]. An investigation applying fecal material transfer from CSDS mice to antibiotics-pretreated mice demonstrated that anhedonia can be transferred via the microbiome [49]. From the 16SrRNA analysis, the authors concluded that *Lactobacillus intestinalis* and *L. reuteri* are responsible for the transferability. Two weeks of treatment with both strains resulted in an anhedonia- and depression-like phenotype in the mice that was blockable by vagotomy. Nevertheless, it might have been that the probiotics mixture in our experiments was at a too low concentration or that the choice of treatment period and included organisms did not allow for the establishment of a beneficial effect. At least when administered in yoghurt as a food carrier in humans, a level of around 10^7^ CFU/mL seems to be needed to provide therapeutic effects [50,51]. As the intestinal system of rodents is not fully comparable to those of humans, and we applied the 10^9^ CFU/mL probiotics within the drinking water and not within food, it cannot be ensured that the chosen amount of bacteria was optimal.

Rodent and human gut and the inhabitant microbiome share many similarities but also sufficient dissimilarities to stay critical about the potential for translation [52]. It has to be noted that a small study on humans with chronic stress revealed a decrease in plasma inflammatory markers such as soluble fractalkine after pretreatment with *Lactiplantibacillus plantarum* HEAL9 for 4 weeks and exposure to acute stress [53]. In regard to the high impact the immune system has on depressive disorders in humans (reviewed in [54]), the role of such probiotics in the treatment of depressive-like behavior in humans should still be further investigated—even if the rodent models give ambiguous results. Moreover, other strains might be more efficient as treatments or as targets to counterbalance stress-induced immune activation. For example, subcutaneous vaccination with Mycobacterium vaccae promoted active stress coping in C57BL6/N mice when given before stress exposure [55]. Comparably, treatment with heat-inactivated *B. breve* ameliorated the SI deficit and suppressed an increase in IL1-b in the prefrontal cortex and hippocampus of male C57BL6/J mice [56].

Despite the lack of influence on the manipulation of the gut microbiome in the CSDS outcome, we nevertheless observed a tremendous activation of enteric neurons in mice subjected to chronic social stress as shown by Arc-dependent staining. As the enteric neurons and the microbiome exert a mutual interplay, an impact on the gut commensals by such stress is conceivable. For example, IL-18 produced by murine enteric neurons but not by epithelial or immune cells are protected against *Salmonella typhimurium* infection by for example, orchestrating defensive peptide secretion by goblet cells [57]. Arc upregulation within distinct brain regions such as the hippocampus has been associated with chronic stress in rodents before (e.g., [58]) and has been even used as a technical tool to investigate expression patterns of resilient and susceptible mice [8]. Its role in enteric neurons, especially under chronic stress, has not been investigated yet. In irritable bowel disease model rats, increased ephB2 expression and synaptic plasticity has been observed recently [59]. EphB2 signaling subsequently led to elevated expression of synaptic-plasticity-related early immediate genes, including Arc. Therefore, activation of Arc expression due to different stressors such as an elevated inflammatory state is conceivable. Increased activity of this immediate early gene under chronic social stress allows speculating on the contribution of the enteric nervous system to microbial changes, but might also be the consequence of those. Conventionalization of germ-free mice with *Bacteroides thetaiotaomicron*, for example, normalized neurotransmitter signaling and increased neuronal budding in the myenteric plexus [60]. Further studies will be needed to untangle microbiome and enteric neurons whether both react separately to stress administration or if one is a consequence of the other. 

While general bacterial counts were not affected as were the two main phyla, Firmicutes and Bacteroidetes, male mice responded with a reduced *Lactobacilli*–*Enterococci* ratio comparing the stressed and control animals. Additionally, *Bacteroides* were found increased and Bacteroidetes/Firmicutes reached nearly statistical significance between resilient and susceptible mice. Reports on microbial changes are highly controversial regarding chronic stress in rodents: for example, susceptible mice have been described to have increased Firmicutes amounts [45] as compared to control animals and resilient individuals. Several reports exist that do not distinguish between resilient and susceptible mice and find, for example, a higher Firmicutes–Bacteroidetes ratio after social defeat [61] but also a lowered Firmicutes amount [12] or unchanged values [10]. Interestingly, the increase in *Bacteroides* that we found for male mice after CSDS was also found in female mice by administering social instability, while the decrease in the *Lactobacillus–Enterococcus* ratio obtained for males could not be confirmed in females. One limitation of comparing the stress paradigms for male and female mice in this study can be seen in the slightly different age of mice at the end of investigation: males were of about 22 weeks old while females were 13–14 weeks old. An earlier developmental stage closer to puberty might be considered as an explanation. However, it has been shown that the microbiome of female rodents seems to be more stable and less vulnerable in this age range as compared to males [62]. Other explanations might be found in the fact that female mice even gained abdominal fat due to the stress paradigm while male mice lost fat deposits, or that the corticosterone levels were still elevated at the time of euthanization [8]. Changes in bacterial amounts such as was found for *Bacteroides* in both sexes might also be relying on effects regarding peristalsis and transit time. For example, women were found to have higher amounts of *Bacteroides* with constipation [63]. As we observed elongated colon tissue in the male mice, a prolonged transit time might—similar to constipation—theoretically explain the increased amounts of *Bacteroides*. However, measurements of colons in female mice and peristalsis measurements did not reveal any changes in this regard (e.g., mean length in controls: 7.28 cm; in stressed mice: 7.35 cm, *p* = 0.73; gut transition time in controls: 201 min, 227 min in stressed mice, *p* = 0.33; data not shown). Recently, not only an increased abundance of *Bacteroides* species was found in major depressive disorder in humans [64,65,66] but also a transferability of negative effects on behavior and neurogenesis by colonization with *B. fragilis*, for example [66]. Moreover, worse depressive symptoms in schizophrenics were associated with Bacteroides [67]. As GABA-producing pathways are actively expressed by *Bacteroides* [68], this capability might be contributing to depressive states in mice and patients or indicate a compensatory attempt of the host–microbiome holobiont, as GABA levels seem to be decreased in depressive patients’ brains [69]. Our study is clearly limited by the fact that we did not characterize the bacterial changes on the species level. However, identification of *Bacteroides* as a microbial genus that seems to increase in both sexes due to two different paradigms of chronic social stress underlines this study’s importance, and further investigation of the influence these commensals might have on depression is needed. The increased levels were restored one year after ending the stress intervention in females, a time point where a partial recovery regarding behavior was also obtained. S-(norketamine) showed anti-depressant-like activity in LPS-treated mice and reduced amounts of two *Bacteroides* strains [70]. Desipramine and aripiprazole also exerted antibiotic effects against *B. fragilis*, for example [71], allowing for speculation if *Bacteroides* might be a valuable target in anti-depressant treatment of present-day stress-induced disorders. Additionally, as resilient and susceptible males here both showed elevated *Bacteroides* levels, it will be of interest to entangle a potential difference in species contributions in both sub groups.

## Data Availability

All data are included in the manuscript.

## Supplementary Materials

The following supporting information can be downloaded at: https://www.mdpi.com/article/10.3390/microorganisms10061077/s1, Figure S1: Activation of enteric neurons during social interaction testing after chronic social instability treatment.
