# Supplementary material for: Resilience and the Gut Microbiome: Insights from Chronically Socially Stressed Wild-Type Mice
Source: Microorganisms. 2022 May 24;10(6):1077. doi: 10.3390/microorganisms10061077 (PMC9231072; doi:10.3390/microorganisms10061077)
Supplement: Supplementary file 1 [file microorganisms-1693574-supplementary.pdf › microorganisms-1693574-supplementary.pdf]

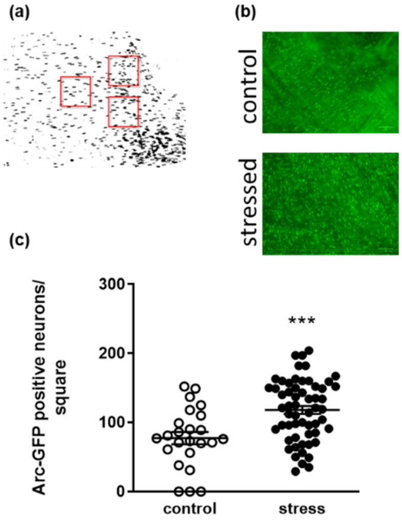

Supplementary Figure S1. Activation of enteric neurons during social interaction testing after chronic social instability treatment. Female Arc-sfGFP mice were treated as described for C57BL6/J mice (see Material and Methods) and analyzed within days after having experienced the last group shuffling. Tamoxifen was injected 9h before social interaction to label activated neurons [8]. Arc-expressing, activated neurons were visualized by their GFP signal under the fluorescent microscope in LMMPs from ileum of stressed and control mice. Stained cells were counted from a defined area (888 x 888 pixel, three areas indicated in red) for each animal (**a**). An exemplary picture for each condition is given in (**b**). (**c**) Statistical analysis was conducted by Student's t-test (n = 24 for control and 58 for stressed animals, derived from five (control) and eleven (stressed) mice per group; ***, $p < 0.0001$).
